# The Beat

**DOI:** 10.1289/ehp.119-a474b

**Published:** 2011-11-01

**Authors:** Erin E. Dooley

## Mercury Levels Lower in Great Lakes, but Still High

Although mercury concentrations have decreased about 20% in fish, fish-eating birds, and inland lake sediments across the Great Lakes region in recent decades, levels in top predator fish and wildlife are still dangerously high, and in some places concentrations in some fish and wildlife may now be increasing, according to a new report.^1^ Levels of mercury in fillets of six commonly consumed game fish species were above the U.S. EPA’s designated human health advisory level of 0.3 ppm across much of the region. The report cited controls on mercury emissions from medical and municipal waste incinerators, chlor-alkali facilities, and other sources as the main reason for decreases in mercury in the region.

**Figure:**
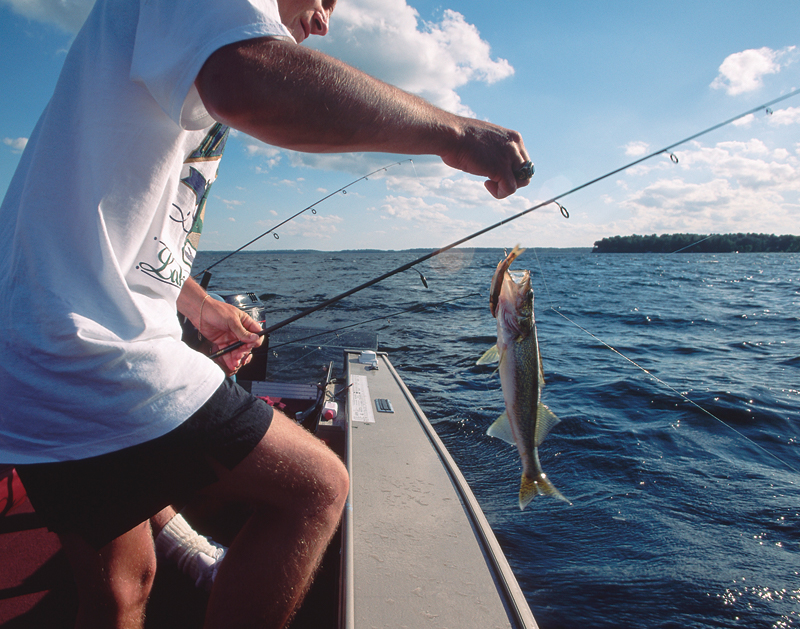
Walleye is one of six Great Lakes region game fish species with consistently high mercury levels. © Phil Schermeister/Corbis

## Sweet Solution for Polluted Soil

Recent news reports bring fresh attention to a sweet ingredient for remediating solvent-laden soils: molasses.^2^ The syrup is diluted and pumped into injection wells from which the solution flows into polluted soil. Once in the soil, the molasses feeds naturally occurring microbes that metabolize solvents into nontoxic by-products. The process has been studied for over a decade and is currently being used in New Jersey and several other states. DuPont recently scratched a pilot study on a similar process that would have used soybean oil to remove perchloroethylene and trichloroethylene from groundwater.^3^

## Dengue Now Endemic in Miami–Dade County

In late September 2011 Florida health officials received confirmation of the second case of locally acquired dengue fever in the Miami–Dade County area for 2011.^5^ The fact that both cases were acquired locally confirms the disease has become endemic in the area. The two cases occurred in widely separated areas within Dade County, so they are probably unrelated (the first case occurred in March). Florida’s Key West experienced several cases of locally acquired dengue in 2009 and 2010, the first time the disease had been seen in the United States since 1934.^6^

**Figure:**
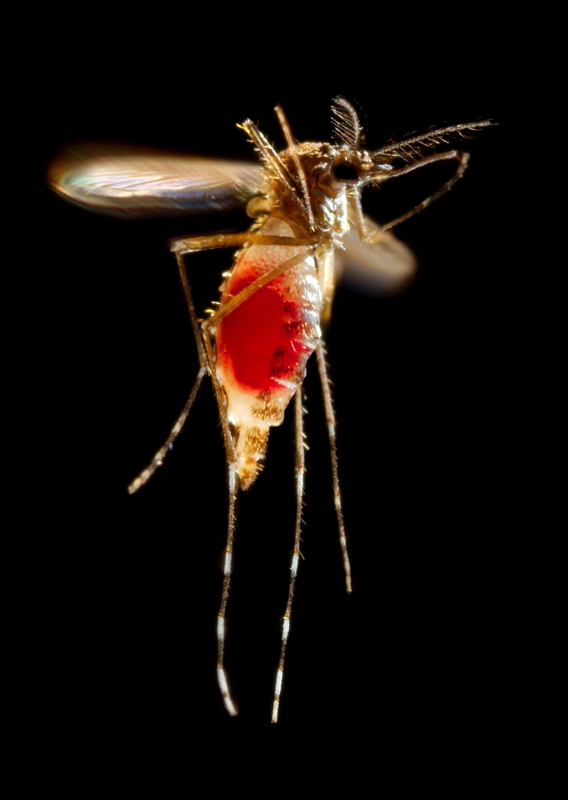
*Aedes aegypti* is the chief mosquito vector of dengue viruses. James Gathany/Centers for Disease Control and Prevention

## BPA and Mammary Tumors in Mice

A new study has found an association between prenatal exposure to low-dose bisphenol A (BPA) and development of mammary tumors in adult female mice.^4^ Mammary epithelial cell numbers were increased to an extent comparable to that seen with diethylbestrol (DES), an agent for which *in utero* exposure has been linked to increased breast cancer risk in humans. BPA, an industrial chemical used to manufacture epoxy resins, polycarbonate plastics, and thermal paper, has not been linked to breast cancer in humans.

## NIEHS to Lead Effort on Climate Change and Human Health

The National Institutes of Health recently announced a new research program to study human health impacts of climate change.^7^ Areas to be addressed include vulnerability to heat-related impacts, changing weather patterns, climate-influenced changes in toxic exposures, and adverse health effects resulting from climate change adaptation and mitigation efforts. Funded studies will help to develop data, methods, and models to support health impact predictions. The program is led by the National Institute of Environmental Health Sciences and includes support from the National Institute on Aging and the Fogarty International Center. Eight grants have been funded.

**Figure:**
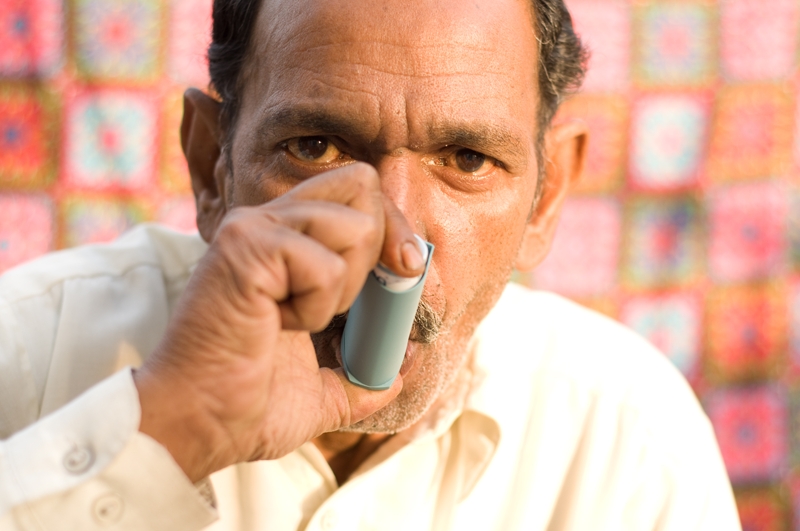
New studies will help refine predictions of health impacts of climate change. Shutterstock.com
